# A randomised, investigator-initiated, clinical trial of the effects of fentanyl on P2Y12-receptor inhibition in patients with ST-elevation myocardial infarction who are pre-treated with crushed ticagrelor: rationale and design of the Opioids aNd crushed Ticagrelor In Myocardial infarction Evaluation (ON-TIME 3) trial

**DOI:** 10.1007/s12471-019-1241-6

**Published:** 2019-02-14

**Authors:** A. H. Tavenier, R. S. Hermanides, J. P. Ottervanger, S. Rasoul, R. J. Slingerland, R. Tolsma, S. van Workum, E. Kedhi, A. W. J. van ’t Hof

**Affiliations:** 1 Department of Cardiology, Isala, Zwolle, The Netherlands; 2 Department of Cardiology, Maastricht University Medical Centre, Maastricht, The Netherlands; 3 Department of Cardiology, Zuyderland Medical Centre, Heerlen, The Netherlands; 4 Department of Clinical Chemistry, Isala, Zwolle, The Netherlands; 5 RAV IJsselland, Zwolle, The Netherlands; 6 RAV Zuid-Limburg, Heerlen, The Netherlands

**Keywords:** ST-elevation myocardial infarction, Ticagrelor, Platelet inhibition, Primary percutaneous coronary intervention, Fentanyl

## Abstract

**Background:**

Fast and accurate platelet inhibition is an important therapeutic goal in the acute treatment of patients with ST-elevation myocardial infarction (STEMI). Platelet inhibitory effects induced by oral P2Y12-receptor antagonists are delayed in STEMI patients undergoing primary percutaneous coronary intervention (PCI) due to haemodynamic changes and delayed gastro-intestinal absorption. Concomitant use of opioids, although recommended in the American College of Cardiology/American Heart Association and European Society of Cardiology STEMI guidelines, further delays gastro-intestinal absorption. To date, trials investigating alternative analgesics in STEMI patients have been scarce. This trial aims to assess the feasibility of a novel drug strategy for treatment of STEMI patients with crushed ticagrelor in combination with paracetamol (acetaminophen) instead of opioids.

**Hypothesis:**

STEMI patients who are pre-treated with crushed ticagrelor and paracetamol have a higher level of platelet inhibition after primary PCI than patients pre-treated with crushed ticagrelor and fentanyl.

**Study design:**

The Opioids aNd crushed Ticagrelor In Myocardial infarction Evaluation (ON-TIME 3) trial is a randomised controlled trial designed to examine whether administration of paracetamol instead of fentanyl can optimise platelet inhibition in STEMI patients who are pre-treated with crushed ticagrelor in the ambulance. One hundred and ninety patients with STEMI will be randomised (1:1 fashion) to intravenous (IV) fentanyl or IV paracetamol. The primary endpoint is the level of platelet reactivity units measured immediately after primary PCI.

**Summary:**

The ON-TIME 3 trial (NCT03400267) aims to achieve optimal platelet inhibition and pain relief in STEMI patients receiving crushed ticagrelor in the ambulance by investigating IV fentanyl and IV paracetamol as analgesics.

## Background

Adequate and optimal platelet inhibition is an important therapeutic goal in the treatment of patients with ST-elevation myocardial infarction (STEMI). Platelet inhibitory effects induced by potent oral P2Y12-receptor antagonists like ticagrelor are delayed in STEMI patients undergoing primary percutaneous coronary intervention (PCI) [1]. STEMI-induced selective shunting of blood to vital organs decreases gastro-intestinal perfusion and induces impaired drug absorption. Furthermore, analgesic drugs such as fentanyl and morphine, which are recommended according to American College of Cardiology/American Heart Association (ACC/AHA) and European Society of Cardiology (ESC) STEMI guidelines [2, 3], are used in STEMI to reduce pain-associated sympathetic activation, which increases vasoconstriction, blood pressure and heart rate [4]. In addition, the uptake of P2Y12 inhibitors can be further delayed by drug interactions. In particular, morphine delays the intestinal drug absorption of P2Y12 inhibitors [5].

In the past, different trials have investigated the influence of morphine on platelet inhibition. The IMPRESSION trial [5] found that administration of morphine in patients with STEMI and non-STEMI influenced the pharmacokinetics by lowering the total exposure to in-hospital-administered ticagrelor and its active metabolite by 36% and 37%, respectively, and delayed the time to maximal plasma concentration of ticagrelor. Also, the effects of morphine on pharmacodynamics have been described and, more frequently, a high platelet reactivity was observed in STEMI patients treated with a P2Y12 inhibitor and receiving morphine [5, 6], which is a phenomenon known to be associated with acute stent thrombosis [7, 8]. The PACIFY trial [9] investigated the influence of fentanyl, a potent opioid, on platelet inhibition and showed lower plasma concentrations of ticagrelor and delayed antiplatelet effects in patients receiving fentanyl during elective angiography.

Different studies have shown that morphine may have adverse effects on inhibitory properties of antiplatelet agents [10–12], myocardial reperfusion [13, 14], and also on all-cause mortality in patients with acute myocardial infarction [15]. However, reports from other studies failed to show such an association [16]. Finally, the pain-relieving effects of morphine in STEMI patients have not been fully investigated yet. To date, trials investigating alternative analgesics in STEMI patients have been scarce.

Different routes of administration of P2Y12 inhibitors like ticagrelor in STEMI patients have been investigated to improve gastro-intestinal absorption. The MOJITO study [17] randomised patients with STEMI undergoing primary PCI into crushed or integral tablets of ticagrelor. Crushed ticagrelor tablet administration in STEMI patients was feasible and provided earlier platelet inhibition compared with standard integral tablets. However, sublingual crushed administration of ticagrelor did not further improve the uptake in patients with unstable angina [18].

The Opioids aNd crushed Ticagrelor In Myocardial infarction Evaluation (ON-TIME 3) trial aims to study the impact of an alternative non-opioid analgesic, paracetamol (acetaminophen), as compared to fentanyl in combination with crushed ticagrelor administration in the ambulance on platelet inhibition in STEMI patients.

## Methods

### Study design and objective

The ON-TIME 3 trial is a prospective, randomised trial, of which the primary objective is to assess the level of platelet inhibition after primary PCI in STEMI patients who are pre-treated with crushed ticagrelor and paracetamol compared to patients who are pre-treated with crushed ticagrelor and fentanyl. The study will be performed in two hospitals: Isala Hospital in Zwolle and Zuyderland Medical Centre in Heerlen. All study drugs are administered in the ambulance.

### Trial registration

This study will be conducted in accordance with the Declaration of Helsinki (64th WMA General Assembly, Fortaleza, Brazil, October 2013), the Medicinal Research Involving Human Subjects Act (WMO) and ICH-Good Clinical Practice (GCP). The study protocol and patient informed consent are approved by the local ethics committee. The ON-TIME 3 trial is registered on ClinicalTrials.gov (NCT03400267).

### Study protocol, randomisation and follow-up

**Fig. 1:**
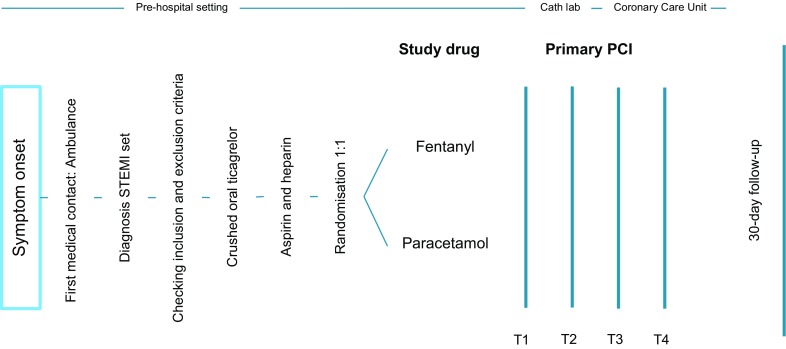
Schematic chart of ON-TIME 3 trial with randomisation and time of measurements (*T1* arrival at cath lab, *T2* end of primary PCI, *T3* 1 h post-primary PCI, *T4* 6 h post-primary PCI). *STEMI* ST-elevation myocardial infarction, *Primary PCI* primary percutaneous coronary intervention

All STEMI patients (defined as on-going chest pain > 30 min and <12 h duration and ST-segment elevation > 0.1 mV in at least two contiguous leads) as diagnosed by the paramedic team on arrival at the patient site that comply with the inclusion and exclusion criteria (Tab. 1) will be asked to participate in this trial. After verbal informed consent patients will be randomised in a 1:1 fashion to either intravenous (IV) fentanyl or IV paracetamol (Fig. 1). The trial is not blinded. All patients will be pre-loaded in the ambulance with unfractionated heparin 5,000 IU and aspirin 500 mg IV according to standard care and 180 mg crushed oral ticagrelor. The paramedic team will register vital parameters and pain scores on a 10-step pain scale. Descriptive statistics will be derived from electronic medical clinical records and stored in an electronic case report form (eCRF). Haemodynamic parameters and data on intensity of pain will be collected in the ambulance at initial presentation. Data on intensity of pain and data on platelet inhibition, including pharmacokinetics and pharmacodynamics, will be collected before (T1) and immediately after primary PCI (T2) at the catheterisation laboratory, 1 h (T3), 3 and 6 h (T4) post-primary PCI at the coronary care unit. A 30-day post-randomisation follow-up will be performed by telephone interview (Fig. 1). Pharmacokinetics will be evaluated using appropriate pharmacokinetics modelling software (e. g. NONNEM or MWPharm) by determination of the concentration of ticagrelor and its active metabolite, AR-C124910XX, using liquid chromatography-mass spectrometry in the clinical chemistry laboratory in Zwolle. Pharmacodynamics will be assessed by a VerifyNow point of care test that measures the platelet reactivity units (PRU). Patients will be treated during hospital admission according to current ACC/AHA and ESC STEMI guidelines [2, 3]. Written informed consent will be obtained during hospital admission.

**Table 1:** Inclusion and exclusion criteria

Inclusion criteria	Exclusion criteria
STEMI patients	Presenting with cardiogenic shock; defined as: systolic blood pressure < 90 mm Hg and heart rate > 100/min and peripheral oxygen saturation < 90% (without oxygen administration)
Age ≥ 18 years	Patient with a nasogastric tube in situ or requiring a nasogastric tube
Symptom onset to STEMI diagnosis (>30 min but <12 h)	Patients who have already received fentanyl or paracetamol < 2 h prior to randomisation
On-going chest pain with pain score ≥ 4 (out of 10-step pain score)	Patients on current treatment with P2Y12 inhibitors (ticagrelor, clopidogrel or prasugrel)
	Patients allergic to fentanyl or paracetamol
	Patients with recent major bleeding complications or contraindication to dual antiplatelet therapy: hypersensitivity to aspirin or ticagrelor, current use of (new) oral anticoagulation, history of bleeding diathesis or known coagulopathy, active bleeding, refusal of blood transfusions, history of intracerebral mass, aneurysm, arteriovenous malformation, or haemorrhagic stroke, and/or known severe liver dysfunction
	Patients who have received any organ transplant or are on a waiting list for any organ transplant
	Patients undergoing dialysis
	Pregnant or lactating females
	Patients currently participating in another investigational drug or device study

*STEMI* ST-elevation myocardial infarction

### Endpoints

#### Primary endpoint

The primary endpoint of the study is the level of PRU measured immediately post-primary PCI or 1 h post-angiography (Tab. 2). Blood will be obtained for our primary endpoint just before sheath removal (T2).

**Table 2:** Primary, secondary and exploratory endpoints

Primary endpoint	Secondary endpoint	Exploratory endpoints (at 30 days of follow-up)
Level of PRU measured immediately post-primary PCI or 1 h post-angiography (T2)	Pain reduction on a 10-step pain scale immediately post-primary PCI or 1 h post-angiography (T2)	MACE (defined as cardiac death, non-fatal re-myocardial infarction or target vessel revascularisation)
	Level of PRU at other time points (T1, T3, T4)	Stent thrombosis (ARC criteria [19])
	High on platelet reactivity defined as PRU > 208 at all time points (T1–4)	Myocardial infarction after PCI [20]
	Area under the curve of the concentrations of ticagrelor and its active metabolite at all time points (T1–4)	Non-CABG-related major bleeding (BARC 3 and 5 criteria [21])
	TIMI 3 flow in the culprit vessel after primary PCI	All-cause mortality

*ARC* Academic Research Consortium, *BARC* Bleeding Academic Research Consortium, *CABG* coronary artery bypass grafting, *MACE* major adverse cardiac events, *PCI* percutaneous coronary intervention, *PRU* platelet reactivity units, *T1* arrival at cath lab, *T2* end of primary PCI, *T3* 1 h post-primary PCI, *T4* 6 h post-primary PCI, *TIMI* thrombolysis in myocardial infarction

#### Secondary endpoints

The secondary endpoints (Tab. 2) consist of the pain reduction on a 10-step pain scale immediately post-primary PCI, the level of PRU at other time points, high on platelet reactivity (HPR) defined as PRU > 208 at all time points, the area under the curve of the concentrations of ticagrelor and its active metabolite at all time points, and thrombolysis in myocardial infarction (TIMI) 3 flow in the culprit vessel after primary PCI. Exploratory safety endpoints are major adverse cardiac events, defined as cardiac death, non-fatal re-myocardial infarction or target vessel revascularisation, stent thrombosis (ARC criteria [19]), myocardial infarction after PCI [20], non-coronary-artery-bypass-grafting (CABG)-related major bleeding (BARC 3 and 5 criteria [21]) and all-cause mortality at 30 days of follow-up.

### Study drugs

Crushed ticagrelor (180 mg) will be prepared by using a crusher cup (Livsane), which allows preparation of crushed ticagrelor in an average time of 1–2 min. After 2 times 5 rotations of the crushing mechanism, the crushed pill contents will be mixed with 25 ml of H_2_O for 30 s in a 150-ml dosing cup. The syringe crusher will be rinsed using an additional 25 ml of H_2_O and will be added to the dosing cup for a total of 50 ml suspension, which will be administered orally. When vomiting occurs and ticagrelor has been spit out, the patient will receive another loading dose of ticagrelor at the catheterisation laboratory. Paracetamol and fentanyl will be intravenously administered. Paracetamol 10 mg/ml is available in ampules of 100 ml (1000 mg) and its infusion time is 15 min. Fentanyl 50 µg/ml is available in 2 ml ampules (100 µg) and will be titrated based on the weight of the patient. Its infusion time is 30 s. The inhibitory pharmacodynamic or pharmacokinetic effects of paracetamol on platelet inhibition have not yet been described in the literature [22–24]. The use of all other intracoronary medication (heparin, nitroglycerin, adenosine and verapamil), the procedural technique at coronary angiography and the type of drug-eluting stent will be at the discretion of the operator. Administration of glycoprotein IIb/IIIa-receptor inhibitors will be restricted to bail-out situations only.

### Pre-specified subgroups for analysis

If a patient randomised to the paracetamol arm experiences unbearable pain (score > 8 on a 10-step pain scale), bail-out medication like fentanyl 50 µg (1 ml) can be given. Patients with bail-out use of fentanyl will be registered and analysed as a subgroup. Also patients with vomiting will be analysed as a subgroup.

### Statistical considerations

This trial is powered for the primary endpoint. Analyses will be performed for both the intention-to-treat and per-protocol population. The primary endpoint will be based on an intention-to-treat analysis.

#### Data analysis

Continuous variables in both study arms will be compared using a Student’s t‑test or Mann-Whitney U test depending on the presence or absence of a normal distribution of the data as assessed by the Kolmogorov-Smirnov test. Proportions will be compared by the Chi-square test or a Fisher’s exact test when appropriate. Exploratory endpoints may be underpowered, and hence graphical methods and estimates with 95% confidence intervals will be used for evaluation. An interim analysis at 50% of inclusions is planned to monitor efficacy and safety during study enrolment.

#### Sample size calculation

The ON-TIME 3 is a superiority trial assessing the use of IV paracetamol compared to IV fentanyl in STEMI patients. Since the effects of paracetamol on PRU are not known yet and no identical studies have been performed, an assumption of the sample size was necessary. We partly based our sample size calculation on data of the IMPRESSION trial [5] and PACIFY trial [9, 25]. Assuming a 60 PRU mean difference immediately after primary PCI between the two arms after a pre-hospital crushed ticagrelor loading dose with a standard deviation of 120 PRU, and 10% rate of invalid results due to haemolysis or technical problems, we estimate that 190 patients (95 per arm) will be needed to obtain a 90% power and two-sided alpha of 0.05.

### Expected results

The ON-TIME 3 trial will be performed to show that STEMI patients treated pre-hospital with crushed ticagrelor and paracetamol have a higher level of platelet inhibition immediately after primary PCI than STEMI patients treated with crushed ticagrelor and fentanyl. The results of this trial have potential implications for the pre-hospital treatment of STEMI patients in the future.

## Discussion

The absorption of more potent P2Y12 platelet inhibitors (ticagrelor and prasugrel) is delayed in STEMI patients due to a reduced gastric perfusion and impaired gastric emptying [1]. Opioids have widely been used in daily practice in STEMI patients and are recommended in international guidelines [2, 3], but opioids also seem to impair gastro-intestinal absorption by reducing gut motility and can further delay the onset of the effect of platelet inhibitors [5]. Moreover, nausea and vomiting are more often seen in patients receiving opioids [4, 6], which reduce the uptake of platelet inhibitors. Therefore, a non-opioid analgesic like paracetamol (acetaminophen) may be beneficial in this respect.

Before effective platelet inhibition is reached, time passes and a gap of sub-optimal platelet inhibition exists, which is an important predictor of ischaemic complications, such as stent thrombosis [7, 8]. To accelerate and improve the absorption of platelet inhibitors several possibilities have been investigated, like pre-hospital administration of oral platelet inhibitors, crushed or chewed ticagrelor and IV administration of platelet inhibitors [26–28]. Administration of crushed ticagrelor has been shown to reduce the time to effective platelet inhibition in STEMI patients compared with standard integral tablets [17].

Other analgesics might be an alternative for opioid use in STEMI patients. While non-steroidal anti-inflammatory drugs are known to increase cardiovascular events [29], paracetamol might be a suitable well-known alternative. IV administered paracetamol is effective more quickly than its oral form. Just a few clinically relevant side effects are known. The main side effect is hypotension when administered intravenously [30]. However, gastro-intestinal side effects of paracetamol are less clear.

The ON-TIME 3 trial continues to search for pain relief and fast and optimal platelet inhibition by investigating an alternative analgesic, paracetamol IV, in STEMI patients that all received crushed ticagrelor in a pre-hospital setting. The results of this trial have potential implications for the pre-hospital treatment of STEMI patients in the future and may change ambulance guidelines.

### Limitations

Although we carefully designed our study, some limitations are present. Firstly, the investigated treatment group is a paracetamol arm instead of a placebo arm. IV paracetamol and IV fentanyl are administered in different ways. Therefore, blinding for randomisation to IV paracetamol and IV fentanyl was not possible. Secondly, since the primary endpoint is the PRU value immediately after primary PCI, the number of patients in this trial is too small to test our hypothesis on clinical outcomes. Moreover, the follow-up period of this trial is only a month after randomisation and therefore long-term outcome and conclusions cannot be drawn based on this research, although optimal antiplatelet therapy in the first hours of onset of STEMI and treatment with primary PCI is thought to be essential for long-term outcomes.

## Summary

The ON-TIME 3 trial is a prospective randomised trial which aims to assess whether STEMI patients who are treated pre-hospital with crushed ticagrelor and IV paracetamol have a higher level of platelet inhibition after primary PCI than patients treated with crushed ticagrelor and IV fentanyl.
